# IL-17A in Human Liver: Significant Source of Inflammation and Trigger of Liver Fibrosis Initiation

**DOI:** 10.3390/ijms23179773

**Published:** 2022-08-29

**Authors:** Daria Kartasheva-Ebertz, Jesintha Gaston, Loriane Lair-Mehiri, Estelle Mottez, Tan-Phuc Buivan, Pierre-Philippe Massault, Olivier Scatton, Sebastien Gaujoux, Jean-Christophe Vaillant, Stanislas Pol, Sylvie Lagaye

**Affiliations:** 1 Institut Pasteur, Immunobiologie des Cellules Dendritiques, INSERM U1223, F-75015 Paris, France; 2 Université de Paris Cité, F-75005 Paris, France; 3 Department of Hepatology and Addictology, AP-HP, Groupe Hospitalier Cochin, Université de Paris, F-75014 Paris, France; 4 Institut Pasteur, Centre de Recherche Translationnelle, F-75015 Paris, France; 5 Department of Digestive Surgery, AP-HP, Groupe Hospitalier Cochin, Université de Paris, F-75014 Paris, France; 6 Department of Hepato-Biliary and Pancreatic Surgery and Liver Transplantation, AP-HP Pitié-Salpêtrière Hospital, Medecine Sorbonne Université, F-75013 Paris, France; 7 Centre de Recherche (CDR) Saint-Antoine, INSERM—UMR_S 938/Sorbonne Université, F-75012 Paris, France

**Keywords:** fibrosis, human liver, type 3 inflammation, IL-17A, human liver slice culture

## Abstract

IL-17A is considered to guide liver inflammation and fibrosis. From twenty-two human liver samples of different fibrosis stages (F0 to F4), IL-17A, IL-22, and TGFβ1 protein expression in liver tissue lysates were analyzed. Ten paired samples of liver tissue (F0–F1 stage) and blood from the same patient were used to analyze intrahepatic and blood T-lymphoid IL-17A^+^ cells by flow cytometry. The analyses have been performed regardless of pathology, considering the stage of fibrosis. Human liver tissue was used for the primary human liver slice cultures, followed by subsequent cytokine stimulation and fibrotic markers’ analysis by ELISA. IL-17A production in human liver tissue was significantly higher in the early fibrotic stage compared with the advanced stage. Th17 T cells and, to a lesser extent, MAIT cells were the main sources of IL-17A in both compartments, the liver and the blood. Moreover, the presence of liver Th17IL-17A^+^INFγ^+^ cells was detected in the liver. IL-17A stimulation of human liver slice culture increased the expression of profibrotic and pro-inflammatory markers. IL-17A, secreted by Th17 and MAIT cells in the liver, triggered fibrosis by inducing the expression of IL-6 and profibrotic markers and could be a target for antifibrotic treatment. Further amplitude studies are needed to confirm the current results.

## 1. Introduction

The development of chronic inflammation promotes the progression of liver fibrosis. A special role in this process is now given to interleukine-17A (IL-17A), which ensures, with its partner IL-22, the establishment of type 3 inflammation in hepatic tissue and provides a massive influx of other immune cells, which enhance and maintain fibrosis activation [1]. The pathogenic role of IL-17A has been demonstrated in different etiological causes of liver fibrosis in animal models [2,3]. However, there is some evidence of its protective role in acute liver conditions [4,5].

Stellate cells (HSCs) are the liver’s leading extracellular matrix (ECM) producers. During activation, stellate cells trans-differentiate into myofibroblasts [6], cells with high proliferative and migratory potential. Myofibroblasts migrate to the site of inflammation and increase the expression of mesenchymal markers such as α-SMA or collagen type 1. Multiple signals, such as cytokines INF*γ*, IL-6, TNF-α, IL-1β, and IL-17A [7], matrix metalloproteinases (MMPs), and growth factors such as TGF-β1, are responsible for the activation of HSCs secreted by damaged hepatocytes, liver sinusoidal endothelial cells (LSECs), Kupffer cells, and T cells. At the same time, HSCs stimulated by IL-17A increase secretion of IL-6, TGF-β1, collagen production, and α-SMA expression—markers of HSCs activation [8].

The accumulation of ECM is related to both high production and low degradation of the matrix. Metalloproteinase inhibitors (TIMPs) are overexpressed during fibrogenesis, particularly TIMP-1, which blocks local degradation of interstitial collagen and also directly promotes myofibroblast activation [9]. The development of fibrosis is a balance between the expression of TIMPs and MMPs [10]. MMP-2 is among the metalloproteases constitutively expressed in the liver by activated stellate cells, whereas MMP-9 reflects the inflammatory process in the tissue, and is expressed by Kupffer cells and monocytes [11].

Experimental models of hepatic fibrosis do not accurately reproduce the environment and pathological processes and only partially reproduce the complex and dynamic process of fibrogenesis and its reversibility [12]. Animal models of fibrosis resolve spontaneously and rapidly once the causative agent ceases to act and the liver regenerates [13], which is not always the case for human fibrosis. The ex vivo model of primary human liver slice culture may be an adequate solution. It is a mini-model that closely resembles the organ from which it is prepared, with all cell types present in their original tissue-matrix configuration [14]. It can allow the study of the early processes of fibrosis initiation and more advanced stages.

We aim to study the protein expression of IL-17A in human liver tissue depending on the fibrosis stage, and to determine which immune lymphoid cell populations secrete IL-17A in human liver (F0–F1) and blood samples. We aim to examine whether IL-17A could trigger the expression of inflammatory and fibrotic markers in our ex vivo model of human liver slice culture and thus be the trigger that initiates the fibrotic process in the liver.

## 2. Results

### 2.1. Basic Characteristics of the Available Human Liver and Blood Samples

The first group of human liver samples comprised 22 specimens, which were divided into 3 subgroups according to their METAVIR score: non-fibrotic samples, F0–F1, intermediate fibrosis stage samples, F2, and advanced fibrosis stage samples, F3–F4. The characteristics are presented in Table 1.

The second group of samples contained 10 human liver specimens and 10 circulating blood specimens corresponding to the same patient. They were obtained as part of the “FIBROTHER” study. Of these 10 liver specimens, 5 were obtained during surgical treatment of hepatocarcinoma, 4 of liver metastases from colorectal cancer, and 1 following the surgical treatment of echinococcosis. All samples were non-fibrotic or with portal fibrosis without septa, F0–F1.

All specimens except one were obtained from the peritumoral region. The F0–F1 specimens, although free of fibrosis, are not healthy because of an underlying disease process in the liver tissue.

### 2.2. IL-17A, IL-22, and TGF-β1 Cytokine Expression in Human Liver Tissue

**Figure 1:**
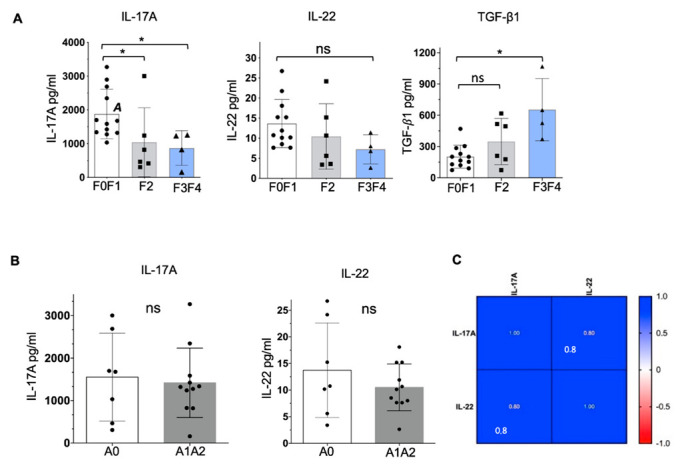
Different IL-17A, IL-22, and TGF-β1 expression in human liver tissue depending on the stage of liver fibrosis. Twenty-two post-surgery human liver samples with different fibrosis stages were lysed, and analyzed by ELISA for IL-17A, IL-22, and TGF-β1. (**A**) IL-17A, IL-22, and TGF-β1 expression in human liver samples at different stages of fibrosis (Kruskal–Wallis test with Dunn’s multiple comparisons correction; * *p* < 0.05; ns: not significant). (**B**) The IL-17A and IL-22 expression in liver tissue was independent of inflammatory activity (Mann–Whitney test; ns: not significant). (**C**) Spearman correlation is significant between IL-17A and IL-22 in human liver lysate samples (ρ = 0.8; *p* < 0.01).

From the first group of human liver samples we measured the protein expression of IL-17A, IL-22, and TGF-β1 directly in human liver lysates by ELISA (Figure 1). Analysis of IL-17A and associated cytokine expression by immune cells extracted from human liver and stimulated in vitro by mitogens for one week, previously, showed a direct correlation of IL-17A and IL-22 secretion with fibrosis stage [3]. In our experiments, IL-17A expression in human liver tissue was significantly higher in non-fibrotic samples compared to late-stage (Figure 1A). The same trend was observed for IL-22. However, as expected, the production of TGF-β1 in liver tissue was correlated with the severity of liver fibrosis (Figure 1A).

There was no significant difference in IL-17A and IL-22 expression depending on the inflammatory activity (Figure 1B). However, there was a significant direct correlation between IL-17A and IL-22 (Figure 1C).

### 2.3. The Profile of Human Intrahepatic T-Lymphoid Immune Cells Differs from That of Blood Cells

**Figure 2:**
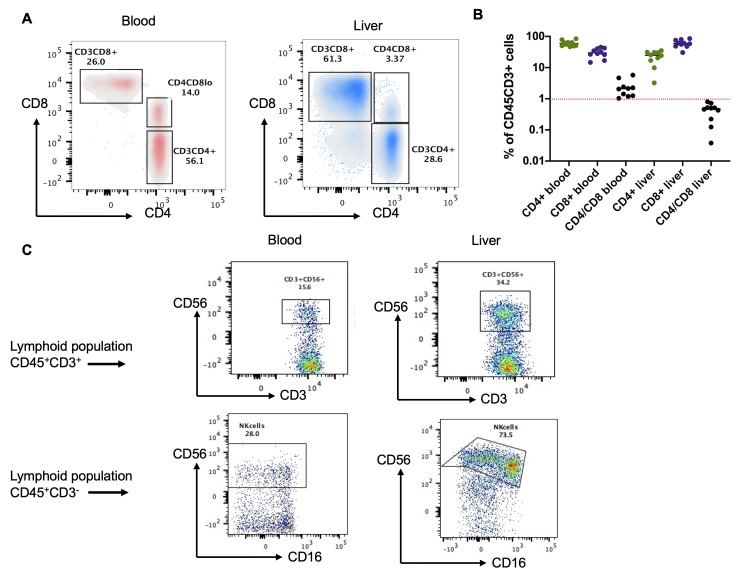
Characterization of lymphoid T cell population in whole blood and liver samples from the FIBROTHER study (*n* = 10). A flow cytometry analysis was performed to characterize the different immune cell types, and the sources of IL-17A in human whole blood and liver samples. (**A**) The main characteristic of immune cells infiltrating the liver is the preponderance of the CD3CD8^+^ population over the CD3CD4^+^. (**B**) The representative graph shows the total distribution of these populations and the CD4/CD8 ratio, i.e., >1 in the blood and <1 in the liver. The graph is representative of a total *n* = 10. (**C**) The liver contains more of the CD45^+^CD3^−^CD56^+^ and CD45^+^CD3^+^CD56^+^CD16^+/−^ cell populations than blood, corresponding to NK cells, respectively.

To characterize the different immune cell types, the sources of IL-17A in human blood and liver, a flow cytometry analysis was performed. Immune cells extracted from the human liver samples and the whole blood of the FIBROTHER study (*n* = 10) were stimulated overnight, following analysis by flow cytometry. The profile of human intrahepatic T-lymphoid immune cells differs from that of the blood. The preponderance of CD45^+^CD3^+^CD8^+^ T lymphocytes over CD45^+^CD3^+^CD4^+^ T lymphocytes was a characteristic feature of intrahepatic immune cells (Figure 2A). Their CD4^+^/CD8^+^ ratio was greater than that of the blood samples and less than that of the liver (Figure 2B). In addition, the liver sample was enriched by particular populations of CD3^+^ and CD3^−^ lymphocytes, including CD56^+^CD161^+^ lymphoid cells, such as NKT, MAIT (mucosal-associated invariant T cells), LT (CD3^+^) (Figure 2C), and NK (CD3^−^) (Figure 2C).

### 2.4. Th17 T Cells Are the Main Source of IL-17A in the Human Liver

**Figure 3:**
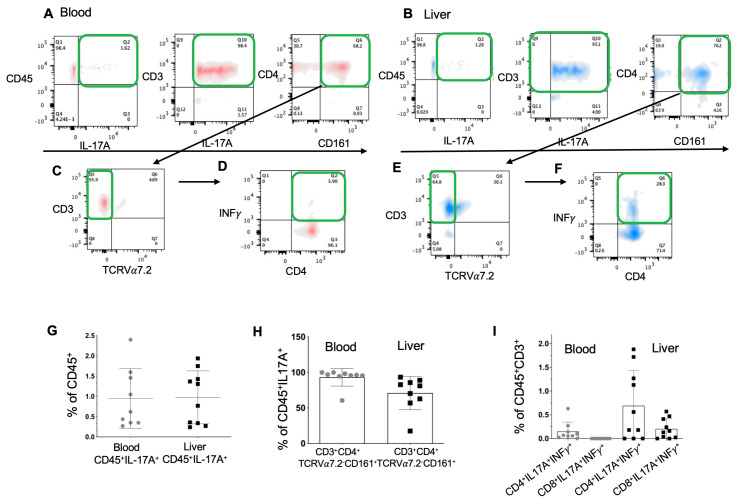
Th17 and MAIT cells are the two main sources of IL-17A in the liver. Representative plots of lymphoid IL-17A expression in patients’ (**A**) blood and (**B**) liver immune cells. Lymphoid IL-17A expression, especially by Th17 (CD45^+^CD3^+^CD4^+^CD161^+^TCRValpha7.2^−^) and MAIT (CD45^+^CD3^+^CD161^+^TCRValpha7.2^+^) cells, in (**C**) blood and (**E**) liver immune cells. Percentage of INFγ+ cells among Th17 IL-17A^+^ cells in the (**D**) blood and (**F**) liver immune cells. (**G**) The percentage of the lymphoid population secreting IL-17A in blood and liver. (**H**) The percentage of IL-17A^+^ cells secreted by CD45^+^CD3^+^CD4^+^TCRVapha7.2^−^ (Th17, Th1) cells in blood and liver samples. (**I**) Percentage of IL-17A^+^INFγ^+^ double-positive cells among Th17 cells of both CD4^+^ and CD8^+^ groups. Presence of CD8^+^IL-17A^+^INFγ^+^ Tc17 cells only in the liver samples (FIBROTHER study).

We studied the immune lymphoid populations that secrete IL-17A in the blood (Figure 3A) and the liver (Figure 3B). The percentage of CD45^+^IL-17A^+^ lymphoid cells was the same in both compartments (Figure 3G). Th17 T cells (CD45**^+^**CD3**^+^**TCRVα7.2^−^CD161**^+^**CD4**^+^**) were the main source of IL-17A in both compartments (Figure 3C,E,H). In the liver, Th17 IL-17A^+^INFγ^+^ cells were present in the liver (Figure 3F), while in the blood (Figure 3D), their frequency was very low/absent. In addition, the Th17 IL-17A^+^INFγ^+^ cell population was represented only by CD4^+^IL-17A^+^INFγ^+^ lymphocytes in the blood, while in the liver, Th17 IL-17A^+^INFγ^+^ lymphocytes were found in two clusters, CD4^+^ and CD8^+^ (Figure 3I). The Th17 T cells in the CD8^+^ cluster were called Tc17 cells [15].

### 2.5. The Human Liver Is Infiltrated by MAITs and T*γδ* Cells

**Figure 4:**
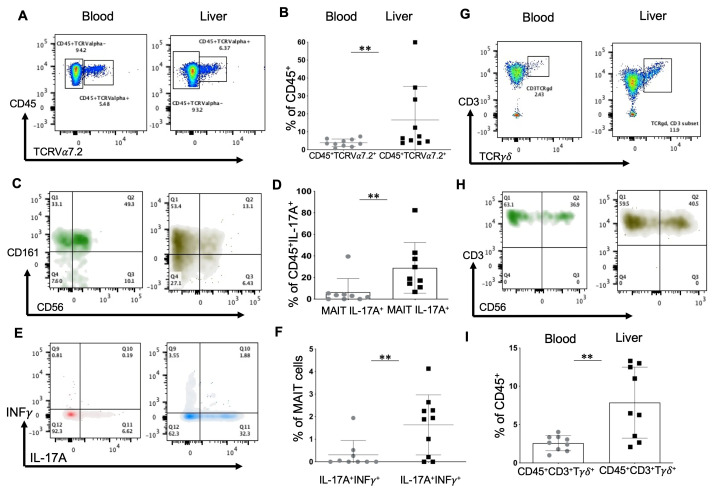
Human liver infiltration by MAIT and Tγδ cells. (**A**) Representative flow cytometry plots of CD45TCRValpha7.2^+^ cells (MAIT cells) in blood and liver samples, (**B**) graphical representation, (**C**) their phenotype, (**E**) their secretion of IL-17A and INFγ. (**D**) Percentage of IL-17A^+^MAIT cells in the CD45^+^IL-17A^+^ lymphoid population in blood and liver samples. (**F**) Graphical representation of the proportion of IL-17A and INFγ double-positive cells among MAIT cells in blood and liver samples. (**G**) Representative flow cytometry plots of Tγδ cells (CD45^+^CD3^+^TCRγδ^+^), along with CD56 surface marker expression (**H**) in blood and liver. (**I**) Graphical representation of Tγδ cells (%) among CD45^+^ lymphoid cells in blood and liver (Mann–Whitney test, ** *p* < 0.01).

When non-canonical T cell populations were analyzed, a relatively high content of liver MAITs (mucosal-associated invariant T cells) was observed (Figure 4A,B). MAITs are innate type T lymphocytes, expressing a semi-invariant TCRVα7.2-Jα33, activated by bacterial metabolites derived from riboflavin synthesis. There are different phenotypes. The two main ones are defined by CD56^+^ expression (Figure 4C), a marker of cell activation and cytotoxicity. The liver was richer in MAITs than the blood (Figure 4B), which is confirmed by other research groups [16], but CD56^+^ MAITs were not preponderant in the liver (Figure 4C). Apart from Th17 and Tc17 T cells, MAIT cells (CD45^+^CD3^+^TCRValpha7.2^+^CD161^+^) secreted IL-17A in the liver (Figure 4D). We also noticed the presence of MAITs that were double-positive for IL-17A and INFγ (Figure 4E,F).

The T*γδ* cells, which are often considered the link between innate and adaptive immunity [17], were prevalent in the liver compared to the blood (Figure 4G,I). Their phenotype was defined as CD45^+^CD3^+^TCR*γδ*^+^CD4^−^CD8^−^. The CD56 marker was also expressed, showing their state of activation (Figure 4H). 

### 2.6. INF*γ* Is Mainly Produced in the Liver by the Canonical Lymphatic Populations (CD3^+^CD4^+^/CD3^+^CD8^+^) as Well as MAITs and T*γδ* Cells

**Figure 5:**
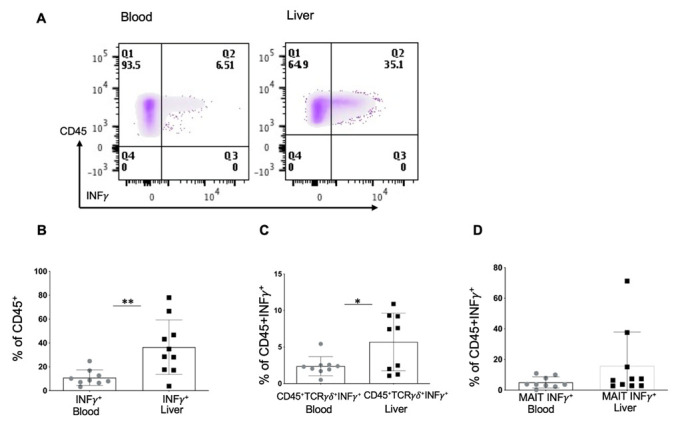
INFγ production in blood and liver samples by CD45^+^ lymphoid population. Representative plots (**A**) and graph (**B**) of lymphoid INFγ production in patients’ blood and liver (percentage (%) of CD45^+^ lymphoid population). (**C**) Percentage (%) of Tγδ cells in CD45^+^INFγ^+^ lymphoid population in liver and blood. (**D**) Percentage (%) of MAIT cells in CD45^+^INFγ^+^ lymphoid cells in liver and blood (Mann–Whitney test, * *p* < 0.05, ** *p* < 0.01).

Among the CD45^+^INFγ^+^ lymphocyte population, the INFγ production was significantly more abundant in the liver (35%) compared to the blood (6.5%) samples (Figure 5A,B). In the liver samples, INF*γ* production by T*γδ* cells (CD45^+^TCR*γδ*^+^) (Figure 5C) and MAITs (Figure 5D) was around 6% and 15%, respectively, and the main source of INFγ remained canonical populations, cytotoxic CD8^+^ T lymphocytes, and Th1 lymphocytes. Among the ten liver samples, we have observed bimodal INF*γ* production by T*γδ* cells in the liver (Figure 5C). For one group, INFγ production was around 6%, and for the other, 12%. However, no correlation with patient pathology of such bimodal secretion was found in this study. The frequency of Th17IL-17A^+^INFγ^+^, Tγδ, and MAITs populations was low in the blood compared to the liver.

### 2.7. IL-17A Increases the Expression of Profibrotic Markers in the Ex Vivo Model of Human Liver Slice Culture

**Figure 6:**
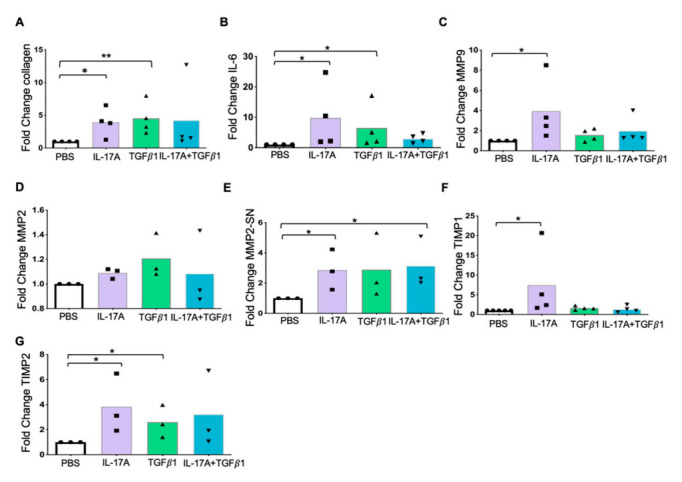
Changes in fibrotic markers’ expression under IL-17A (30 ng/mL) and TGF-β1 (2.5 ng/mL) of F0–F1 human liver slice culture for 50 h (ELISA). The results are presented as fold change-treated/PBS. (**A**) Fold change for collagen expression in liver tissue lysates. (**B**) Fold change for IL-6 expression in liver tissue lysates. (**C**) Fold change for MMP-9 expression in liver tissue lysates. (**D**) Fold change for MMP-2 expression in liver tissue lysates. (**E**) Fold change for activated MMP-2 secretion in the supernatant. (**F**) Fold change for TIMP-1 expression in liver tissue lysates. (**G**) Fold change for TIMP-2 expression in liver tissue lysates. The statistical test for ELISA: one-sample two-tailed t-test for F = log_2_(fold change), * *p* < 0.05, ** *p* < 0.01.

Knowing the advances of IL-17A studies in hepatic fibrosis, we decided to verify whether IL-17A acts on the expression of fibrotic and pro-inflammatory markers in human tissue. The human liver slices culture (F0–F1) was stimulated by either PBS, IL-17A (30 ng/mL), or by TGF-β1 (2.5 ng/mL), or IL-17A+TGF-β1 for 50 h, and the expression of fibrotic markers was analyzed in tissue lysates by ELISA. Stimulation of human liver slices by IL-17A increased the expression of collagen (Figure 6A) and IL-6 (Figure 6B), the two crucial markers of fibrotic processes in the liver. After stimulation by TGF-β1, the expression of collagen (Figure 6A) and IL-6 was also increased (Figure 6B). The combination of IL-17A+TGF-β1 did not show any synergistic action on the collagen or IL-6 secretion, but on the contrary, in the case of IL-6, the addition of TGF-β1 decreased the activating effect of IL-17A during the first 50 h of the human liver slice culture. 

Metalloproteinases exist in the liver in two forms, proactive and active. In liver tissue lysates, both forms were detected, while in the culture supernatant, only the active form was detected. Stimulation by IL-17A caused an increase in MMP-9 in the liver tissue lysates (Figure 6C) but showed no effect in the supernatant. For MMP-2, the situation was the opposite: the increase of the active form, under IL-17A stimulation, was detected in the culture supernatant (Figure 6E), but not in the liver tissue lysates (Figure 6D). At this stage, we could assume that IL-17A increased the activation of MMP-2 but did not influence de novo synthesis, whereas for MMP-9, IL-17A acted in the opposite direction. The stimulation of liver slices by IL-17A+TGF-β1 once again showed its inhibitory effect, this time on MMP-9 synthesis (Figure 6C), and no effect on MMP-2 synthesis (Figure 6D). On the other hand, this effect was absent in the case of MMP-2 active forms (Figure 6E). 

The expression of TIMP-1 was induced by IL-17A. Neither TGF-β1 nor the IL-17A+TGF-β1 combination showed any effect (Figure 6F). The TIMP-2 responded to IL-17A+TGF-β1 stimulation (Figure 6G).

Overall, IL-17A increased the secretion of profibrotic markers and IL-6 in human liver slice culture, but the combination of IL-17A+TGF-β1 had some inhibitory effect on the expression of IL-6, MMP-9, and TIMP-1.

## 3. Discussion

In our work, we have measured IL-17A concentration in human liver tissue, finding that it was significantly higher in early-stage fibrosis compared to late-stage. Th17 T cells (CD45**^+^**CD3**^+^**TCRVα7.2^−^CD161**^+^**CD4**^+^**) were the main source of IL-17A in the human liver and blood. The human liver contains a Th17 IL-17A^+^INFγ^+^ population, while their frequency was low in the blood. We observed that the liver was richer in MAIT cells than the blood, as well as in T*γδ* cells. Using the human non-fibrotic liver slice culture model, we showed that IL-17A stimulation increased the expression of profibrotic markers (TGF-β1, collagen) and IL-6 for the first 50 h of the human liver slice culture. 

Despite the literature data showing a correlation between the stage of fibrosis and the level of IL-17A and IL-22 production by intrahepatic immune cells, after an in vitro stimulation by mitogens for one week [3], we observed the opposite results. By measuring the expression of these cytokines directly in human tissue lysates, without stimulation, a higher level of IL-17A was observed in non-fibrotic liver samples or liver samples with early fibrosis. IL-22 levels did not change significantly. There are several possible reasons for this. In our study, IL-17A and IL-22 expression was quantified in human liver tissue lysates after blood lavage, which has never been done before. In 2018, Fabre et al. measured cytokine release by isolated intrahepatic immune cells after several hours or even for one week of in vitro stimulation [3]. Tissue lysates contain parenchyma, connective tissue, and hepatic immune cells, which differ from the situation when only cytokine secretion by purified immune cells is measured in vitro. We cannot exclude the production of IL-17A in the liver by non-immune cells. However, the TGF-β1 concentration measured in tissue lysates followed the severity of fibrosis, which is consistent with all data in the scientific literature [18]. Non-immune liver cells are the most important producers of TGF-β1 [18]. A strong correlation between IL-17A and IL-22 (Figure 1, ρ = 0.8) may indicate their synergistic action in liver tissue. However, to date, most research on IL-22 has focused on its protective, anti-fibrogenic aspect [19,20]. Probably, its action does not depend directly on the stage of fibrosis but on the state of the causal disease, because IL-22 is also known to promote the development of hepatocarcinoma [21].

Access to human liver samples is quite complicated. Most liver diseases are not surgical diseases. Liver specimens outside of transplantation are available from hepatocarcinoma, cholangiocarcinoma, or liver metastasis surgery. These are peritumoral tissue specimens with traces of underlying diseases (NASH, viral hepatitis, alcohol). Additionally, liver samples are available in the case of rare parasitic diseases such as echinococcosis. Samples may also be obtained as a result of liver transplantation, which is performed in 50% of cases for cirrhosis, primary liver tumors (17%), cholestatic disease (10% of all transplants), acute liver failure of various causes (9.1%), and metabolic disease (6%) [22]. A healthy liver is only available in the context of a graft, which was not the case in this study. The difficulty may be that all these pathologies may themselves cause increased IL-17A production, regardless of the fibrosis stage. IL-17A plays a role in antiparasitic immunity [23], and in metastatic colon cancer [24], one of the most common cancers with liver metastases. NASH is also suspected of being linked to IL-17A [25,26]. Thus, although IL-17A is associated with and contributes to fibrotic events in the liver, this is not its exclusive and perhaps not its most important functional role. Moreover, fibrosis is in constant equilibrium: it can be activated and remodeled depending on tissue environmental factors. The distribution of fibrotic changes in the liver is not homogeneous. Additionally, given the reversibility of fibrotic processes, there are patients with regressed fibrosis whose liver tissue condition cannot be equated with the definition of a healthy liver. The F1 group may contain specimens with some level of chronic inflammation and, consequently, high IL-17A and IL-22 concentrations in the tissue. All these factors and differences could influence our results.

The FIBROTHER study allowed us to describe the type of human immune cells that secrete IL-17A in the blood and the liver. We obtained a global view of IL-17A production in the two compartments. For the moment, 10 human liver non-fibrotic samples with underlying diseases of various etiologies did not permit us to characterize the immune composition according to the fibrosis stage. Nevertheless, the liver specimens under study were not healthy, even if they were not fibrotic, which may have affected the immune phenotype.

The production of IL-17A in the liver and blood is performed mainly by Th17 cells. Although IL-17A secretion in the blood is ensured only by Th17 cells, and in the liver Th17/Tc17 and MAIT cells can secrete it, INFγ is capable of inhibiting IL-17A secretion [27]. However, the liver contains Th17 and Tc17 cells that are double-positive for IL-17A and INFγ, indicating the plasticity of these populations. A similar situation has occurred with MAIT cells: the liver contained double-positive MAIT INFγ^+^IL-17A^+^ cells, while there were almost none in the blood. There are very few data in the literature on the liver’s double-production of INFγ and IL-17A by T cells. Based on the analysis of the available data, we can speculate that these cells have increased cytotoxicity [28], but this requires further investigation.

We observed that a group of liver samples reached more than 10% of T*γδ* lymphocytes (Figure 4I), while a healthy liver contains about 3% [17,29], with a bimodal production of INF*γ* (Figure 5C). This is most likely related to the underlying liver disease. However, we could not find any factor that shares this distribution, with n = 10. Thus, a more significant number of samples will be required.

Most studies on IL-17A and liver fibrosis have been conducted on a mouse model [30,31]. The human case is much more complex due to intra- and inter-individual variability and the frequent presence of underlying disease. A human liver slice culture model can be an excellent tool for this purpose. In this ex vivo model, IL-17A appears as a trustworthy profibrotic trigger, increasing the secretion of the main components of fibrosis—Pro-Collagen1A1, MMP-2 and -9, TIMP-1 and -2, and pro-inflammatory factor IL-6—for the first 50 h of the human liver slice culture. In contrast, the effect of the cytokine combination IL-17A+TGF-β1 tended to inhibit the stimulation induced by IL-17A alone (Figure 6). This effect has been reported on the primary culture of human fibroblasts, where IL-17A, in the presence of TGF-β1, inhibited collagen production, establishing a kind of regulation [32]. This result may be related to the culture itself as an adaptive mechanism. In this case, it will be necessary to increase the duration of the culture. It may also be a self-regulating mechanism, which requires further study. In this work, the stimulation of the human liver slices was carried out for 50 h, which may not be enough time for many biological effects to occur. This duration was chosen for several reasons. We wanted to preserve the possible effect of intrahepatic immune cells. Nevertheless, their lifespan under these culture conditions, unsuitable for immune cells without cytokine support, could not be expected to be longer than 48 h. In addition, the quantity of available tissue did not always allow us to achieve all the desired stimulations, and at the same time, to lengthen the culture. It was also preferable not to lose liver slice viability for these first experiments, which remained 100% viable for 50 h in culture [33].

In addition to the above-mentioned limitations of the study, we would like to highlight the need for further study of human liver tissue with a larger sample. A comparative analysis of the immunological composition of liver tissue in different pathologies at different stages of fibrosis is needed, considering the patient’s treatment. Additionally, our study did not have access to normal healthy liver tissue, which is also unnecessary when comparing pathology with the norm. Further research is needed.

## 4. Materials and Methods

### 4.1. Human Liver Tissue and Blood Specimens

Adult human liver tissue and blood specimens were obtained from selected patients with different liver pathologies. Written informed consent was obtained from each patient included in the study. The experimental procedures were carried out in accordance with French laws and Regulations and ethics committees from Pitié-Salpétrière Hospital, Cochin Hospital, and Pasteur Institute (France).

The first group of liver tissue samples from 22 patients (6 females/16 males) were divided into 3 categories according to their METAVIR score and inflammatory activity grade (Table 1) [34].

The second group of samples consisted of 10 paired liver (F0–F1 stage) and blood samples from the same patient. Intrahepatic immune cells and blood cells from these samples were analyzed by flow cytometry (Etude FIBROTHER: Promoter: Pasteur Institute; N° Promotor: 2018-069; No ID RCB: 2019-A00128-49; N°CPP Est-1: SI 19.02.06.68507; ClinicalTrials.gov: NCT03979417). Tissue samples (F0–F1) from group 2 were also used for the culture of human liver slices.

### 4.2. Human Liver Slice Culture

The liver samples were transported on ice to the laboratory as quickly as possible. Immediately after surgical resection, all liver pieces were collected and stored on ice in a sterile solution from the University of Wisconsin (ViaSpan; Barr Laboratories, Inc., Pomona, NY, USA) until slicing. The time between harvesting and slicing was kept to an absolute minimum (<2 h). The liver slices were prepared and cultured as previously described [14].

### 4.3. Isolation of Intrahepatic and Circulating Immune Cells and Flow Cytometry Analysis

All liver samples were processed on the day they were received. First, the liver samples were infused with 100 mL of HBSS (Hank’s Balanced Salt Solution) to remove as many circulatory cells as possible. Subsequently, the samples were incubated with a 2% collagenase D solution (Roche), cut into small pieces with a surgical scalpel, and left in the infusion solution for 20 min in an oven at 37 °C, with 5% CO_2_. The samples were then diluted on a 70-micrometer cell strainer using a syringe plunger placed on a pre-filled tube with PBS. Then, the cell suspension was centrifuged at 1000 rpm for 2 min to pellet the hepatocytes. The recovered supernatant was washed and then diluted in 40% Percoll, deposited on a gradient (80%, 60%) of Percoll (GE Healthcare, lot 10283278), and centrifuged at 2000 rpm for 20 min without braking. Leukocyte rings (the first between 80% and 60%, the second between 60% and 40%) were recovered, washed, resuspended in PBS 1×, and leukocytes were counted using the Malassez cell. The cells were then stimulated, labeled, and analyzed by flow cytometry.

After overnight stimulation of the whole blood, circulating immune cells were isolated from the blood on a density gradient medium (Ficoll–Paque, GE Healthcare). The blood was diluted 1:1 with PBS 1× and deposited on the gradient and centrifuged at 2000 rpm for 20 min without braking. The leukocyte ring was recovered, washed, resuspended in PBS 1×, and leukocytes were counted using the Malassez cell. The cells were then labeled and analyzed by flow cytometry.

Liver leukocytes were analyzed by flow cytometry on a BD LSR Fortessa™ Flow Cytometer (BD Biosciences). The antibodies used for the cytometry are presented in Supplementary Table S1. Intracellular labeling was performed using the BD Cytofix/Cytoperm kit (BD Biosciences Cat No. 554715) according to the manufacturer’s protocol. The results were analyzed with the FlowJo software. For intracellular staining of blood immune cells, n = 9. Negative controls for intracellular cytokine production are presented in Supplementary Figure S1 and the gating strategy is presented in Figure S2.

### 4.4. Stimulation of Intrahepatic and Circulating Blood Immune Cell Culture

For the flow cytometry analysis, intrahepatic immune cells and circulating blood were stimulated overnight with PMA (0.5 μg/mL) (Phorbol 12-myristate 13-acetate, Sigma Aldrich P81139) and Ionomycin (250 ng/mL) (Sigma Aldrich I3909). Brefeldin A (10 μg/mL) (Sigma Aldrich B6542) was added one hour after the start of the stimulation. Human liver slice culture was stimulated for 50 h with the IL-17A (30 ng/mL) (Life Technologies, 4331182) or TGF-β1 (2.5 ng/mL) (RD system, 7754-BH-005) cytokines alone or in combination.

### 4.5. Cytokines and Fibrosis Markers’ Analysis

The culture supernatants, the lysates of primary liver tissue, and lysates from the primary human liver slice cultures, stimulated or not, were frozen at −80 °C until analysis. The protein expression levels of the cytokines IL-17A, IL-22, and TGF-β1, as well as the protein expression of MMP-2, MMP-9, collagen, IL-6, TIMP-1, and TIMP-2, were analyzed by ELISA (enzyme-linked immunosorbent assay) using the commercial DuoSet ELISA (RD system) kits (Supplementary Table S2) following the manufacturer’s protocols.

### 4.6. Statistics

Statistical tests were carried out with GraphPad Prism 6 and 7 (GraphPad Software, La Jolla, CA). Values are expressed as means ± standard deviation (SD). Differences between the groups (Figure 1) were determined by the Kruskal–Wallis test with Dunn’s correction. Correlations were tested using Spearman’s rank correlation. The Mann–Whitney test was used to compare flow cytometry data. A one-sample two-tailed *t*-test for F = log_2_(fold change) was used to compare the expression of the different proteins analyzed by ELISA (Figure 6).

## 5. Conclusions

Overall, we observed that IL-17A plays its profibrotic and pro-inflammatory role in the culture of F0-F1 human liver slices, inducing the expression of profibrotic markers. The cell profiles producing IL-17A in the patient’s liver and blood were different: Th17 cells in the blood and Th17, Tc17, and MAIT cells in the liver. We have shown the presence of double-positive Th17 INFγ^+^IL-17A^+^ and MAIT INFγ^+^IL-17A^+^ cells in the liver. Meanwhile, at the same time, the concentration of IL-17A in liver tissue was higher either in the early stage of fibrosis or in the total absence of fibrosis. It can be assumed that IL-17A production, while contributing to the development of fibrosis, is not a critical element that defines fibrotic events but is possibly necessary to initiate the profibrotic reaction. Its expression could be related to the pathologies that induce fibrosis, not to the mechanism of fibrosis itself. Nevertheless, the level of Il-17A expression might be a precursor marker of liver fibrosis and could be a target for anti-fibrotic liver treatment.

## Figures and Tables

**Table 1:** Characteristics of the first group of human liver samples.

Fibrosis Stage	F0–F1	F2	F3–F4
Number of samples	12 samples	6 samples	4 samples
Pathology	9 Liver metastasis 1 Benign tumor 2 Hepatocarcinoma	2 Cholangiocarcinoma1 Benign tumor 1 Cancer metastasis of another organ 2 Hepatocarcinoma	1 Cholangiocarcinoma+ hepatocarcinoma 3 Hepatocarcinoma
Activity (A)	A0—4 samples	A0—2 samples	A1–A2—2 samples
	A1–A2—8 samples	A1–A2—4 samples	2 undefined samples

Fibrosis grade: METAVIR scores (F). Necrotic inflammation: activity grade (A). A “significant” fibrosis, as defined by a fibrosis grade (F), is greater than 1 by the METAVIR scoring system. A significant necrotic inflammation, as defined by an activity grade (A), is greater than 1 by the METAVIR scoring system (15). Fibrosis stage: F0: no fibrosis, F1: portal fibrosis without septa, F2: portal fibrosis with few septa, F3: numerous septa without cirrhosis, F4: cirrhosis. Activity grade: A0: no activity, A1: mild activity, A2: moderate activity, A3: severe activity.

## Data Availability

All data associated with this study are present in the paper and/or Supplementary Materials.

## Supplementary Materials

The following supporting information can be downloaded at: https://www.mdpi.com/article/10.3390/ijms23179773/s1.

## References

[1] Kartasheva-Ebertz D.M., Pol S., Lagaye S. (2021). Retinoic Acid: A New Old Friend of IL-17A in the Immune Pathogeny of Liver Fibrosis. Front. Immunol..

[2] Meng F., Wang K., Aoyama T., Grivennikov S.I., Paik Y., Scholten D., Cong M., Iwaisako K., Liu X., Zhang M. (2012). Interleukin-17 Signaling in Inflammatory, Kupffer Cells, and Hepatic Stellate Cells Exacerbates Liver Fibrosis in Mice. Gastroenterology.

[3] Fabre T., Molina M.F., Soucy G., Goulet J.-P., Willems B., Villeneuve J.-P., Bilodeau M., Shoukry N.H. (2018). Type 3 cytokines IL-17A and IL-22 drive TGF-β-dependent liver fibrosis. Sci. Immunol..

[4] Wondimu Z., Santodomingo-Garzon T., Le T., Swain M.G. (2010). Protective Role of Interleukin-17 in Murine NKT Cell-Driven Acute Experimental Hepatitis. Am. J. Pathol..

[5] Acharya D., Wang P., Paul A.M., Dai J., Gate D., Lowery J.E., Stokic D.S., Leis A.A., Flavell R.A., Town T. (2017). Interleukin-17A Promotes CD8+ T Cell Cytotoxicity To Facilitate West Nile Virus Clearance. J. Virol..

[6] Hernández-Gea V., Ghiassi-Nejad Z., Rozenfeld R., Gordon R., Fiel M.I., Yue Z., Czaja M.J., Friedman S.L. (2012). Autophagy releases lipid that promotes fibrogenesis by activated hepatic stellate cells in mice and in human tissues. Gastroenterology.

[7] Pinzani M. (2015). Pathophysiology of Liver Fibrosis. Dig. Dis..

[8] Tan Z., Qian X., Jiang R., Liu Q., Wang Y., Chen C., Wang X., Ryffel B., Sun B. (2013). IL-17A Plays a Critical Role in the Pathogenesis of Liver Fibrosis through Hepatic Stellate Cell Activation. J. Immunol..

[9] Masuzaki R., Kanda T., Sasaki R., Matsumoto N., Ogawa M., Matsuoka S., Karp S.J., Moriyama M. (2020). Noninvasive Assessment of Liver Fibrosis: Current and Future Clinical and Molecular Perspectives. Int. J. Mol. Sci..

[10] Roeb E. (2018). Matrix metalloproteinases and liver fibrosis (translational aspects). Matrix Biol..

[11] Iredale J.P., Thompson A., Henderson N.C. (2013). Extracellular matrix degradation in liver fibrosis: Biochemistry and regulation. Biochim. Biophys. Acta Mol. Basis Dis..

[12] Perlman R.L. (2016). Mouse models of human disease. Evol. Med. Public Health.

[13] Kisseleva T., Cong M., Paik Y., Scholten D., Jiang C., Benner C., Iwaisako K., Moore-Morris T., Scott B., Tsukamoto H. (2012). Myofibroblasts revert to an inactive phenotype during regression of liver fibrosis. Proc. Natl. Acad. Sci. USA.

[14] Kartasheva-Ebertz D., Gaston J., Lair-Mehiri L., Massault P.-P., Scatton O., Vaillant J.-C., Morozov V.A., Pol S., Lagaye S. (2021). Adult human liver slice cultures: Modelling of liver fibrosis and evaluation of new anti-fibrotic drugs. World J. Hepatol..

[15] Srenathan U., Steel K., Taams L.S. (2016). IL-17+ CD8+ T cells: Differentiation, phenotype and role in inflammatory disease. Immunol. Lett..

[16] Lamichhane R., Munro F., Harrop T.W.R., de la Harpe S.M., Dearden P.K., Vernall A.J., McCall J.L., Ussher J.E. (2021). Human liver-derived MAIT cells differ from blood MAIT cells in their metabolism and response to TCR-independent activation. Eur. J. Immunol..

[17] Hammerich L., Tacke F. (2014). Role of gamma-delta T cells in liver inflammation and fibrosis. World J. Gastrointest. Pathophysiol..

[18] Friedman S.L. (2008). Hepatic Stellate Cells: Protean, Multifunctional, and Enigmatic Cells of the Liver. Physiol. Rev..

[19] Kong X., Feng D., Wang H., Hong F., Bertola A., Wang F.-S., Gao B. (2012). Interleukin-22 Induces Hepatic Stellate Cell Senescence and Restricts Liver Fibrosis. Hepatology.

[20] Wu Y., Min J., Ge C., Shu J., Tian D., Yuan Y., Zhou D. (2020). Interleukin 22 in Liver Injury, Inflammation and Cancer. Int. J. Biol. Sci..

[21] Jiang R., Tan Z., Deng L., Chen Y., Xia Y., Gao Y., Wang X., Sun B. (2011). Interleukin-22 promotes human hepatocellular carcinoma by activation of STAT3. Hepatology.

[22] Müller P.C., Kabacam G., Vibert E., Germani G., Petrowsky H. (2020). Current status of liver transplantation in Europe. Int. J. Surg..

[23] Mezioug D., Touil-Boukoffa C. (2012). Interleukin-17A correlates with interleukin-6 production in human cystic echinococcosis: A possible involvement of IL-17A in immunoprotection against Echinococcus granulosus infection. Eur. Cytokine Netw..

[24] Wu D., Wu P., Huang Q., Liu Y., Ye J., Huang J. (2013). Interleukin-17: A Promoter in Colorectal Cancer Progression. Clin. Dev. Immunol..

[25] Gomes A.L., Teijeiro A., Burén S., Tummala K.S., Yilmaz M., Waisman A., Theurillat J.-P., Perna C., Djouder N. (2016). Metabolic Inflammation-Associated IL-17A Causes Non-alcoholic Steatohepatitis and Hepatocellular Carcinoma. Cancer Cell.

[26] Ernst M., Putoczki T. (2014). IL-17 Cuts to the Chase in Colon Cancer. Immunity.

[27] Harrington L.E., Hatton R.D., Mangan P.R., Turner H., Murphy T.L., Murphy K.M., Weaver C.T. (2005). Interleukin 17–producing CD4+ effector T cells develop via a lineage distinct from the T helper type 1 and 2 lineages. Nat. Immunol..

[28] Tajima M., Wakita D., Satoh T., Kitamura H., Nishimura T. (2011). IL-17/IFN-γ double producing CD8+ T (Tc17/IFN-γ) cells: A novel cytotoxic T-cell subset converted from Tc17 cells by IL-12. Int. Immunol..

[29] Wang Y., Zhang C. (2019). The Roles of Liver-Resident Lymphocytes in Liver Diseases. Front. Immunol..

[30] Delire B., Stärkel P., Leclercq I. (2015). Animal Models for Fibrotic Liver Diseases: What We Have, What We Need, and What Is under Development. J. Clin. Transl. Hepatol..

[31] Bao Y., Wang L., Pan H., Zhang T., Chen Y., Xu S., Mao X., Li S. (2021). Animal and Organoid Models of Liver Fibrosis. Front. Physiol..

[32] Dufour A.M., Alvarez M., Russo B., Chizzolini C. (2018). Interleukin-6 and Type-I Collagen Production by Systemic Sclerosis Fibroblasts Are Differentially Regulated by Interleukin-17A in the Presence of Transforming Growth Factor-Beta 1. Front. Immunol..

[33] Lagaye S., Shen H., Saunier B., Nascimbeni M., Gaston J., Bourdoncle P., Hannoun L., Massault P.-P., Vallet-Pichard A., Mallet V. (2012). Efficient replication of primary or culture hepatitis C virus isolates in human liver slices: A relevant ex vivo model of liver infection. Hepatology.

[34] Bedossa P., Poynard T. (1996). An algorithm for the grading of activity in chronic hepatitis C. The METAVIR Cooperative Study Group. Hepatology.

