# Supplementary material for: IL-17A in Human Liver: Significant Source of Inflammation and Trigger of Liver Fibrosis Initiation
Source: Int J Mol Sci. 2022 Aug 29;23(17):9773. doi: 10.3390/ijms23179773 (PMC9456490; doi:10.3390/ijms23179773)

Supplementary Tables

Table S1: List of antibodies used in the study

Markers	Compagnies	labelling	Clone	Lot
**CD45**	BD	BUV805	HI30	7205769
**CD3**	BD	PE-CF594	UCHT1	9102751
**CD4**	BD	FITC		41807
**CD8**	BD	BV786	RPA-T8	8149724
**CD161**	BD	PerCP	HP-3G10	B235672
**CD196**	BD	PE		2124684
**CD56**	BD	BUV395	NCAM16.2	6055836
**CD16**	BD	PerCP-Cy5.5	3G8	2137566
**TCRgd**	BD	PE		41154
**TCRValpha7.2**	MACS	APC		5190813035
**HLA-DR**	BD	BUV395	G46-6	6119962
**Live-DEAD**	invitrogen	Violet dead		2116142
**CD15**	BD	BV711	W6D3	9206724
**CD14**	BD	PE-Cy7		7250714
**CD11b**	MACS	PerCP-Vio700	REA713	5180611205
Intracellular				
**IL-17A**	BD	BV510	BL168	B262925
**INFγ**	BD	BV650	4S.B3	9142880

Tableau S2: List of Elisa kits used in the study

**Markers**	**Compagnies**	**Catalog N**	**Lot**
MMP-2	RD systems	DY902	P218675
TIMP-2	RD systems	DY971	P182406
MMP-9	RD systems	DY911-05	P217702
TGF-β1	RD systems	DY240	P196660
IL-6	RD systems	DY206-05	P219259
IL-22	RD systems	DY782-05	P103191
Pro-Collagen1A1	RD systems	DY6220_05	P221260
TIMP-1	RD systems	DY970-05	P221002
IL-17A	RD systems	DY317-05	P100117

Supplementary Figures

Figure S1

Negative control for intracellular cytokine production for IL-17A and INFγ

Blood
FMO IL17A/ INFγ

Liver
FMO IL17A/ INFγ

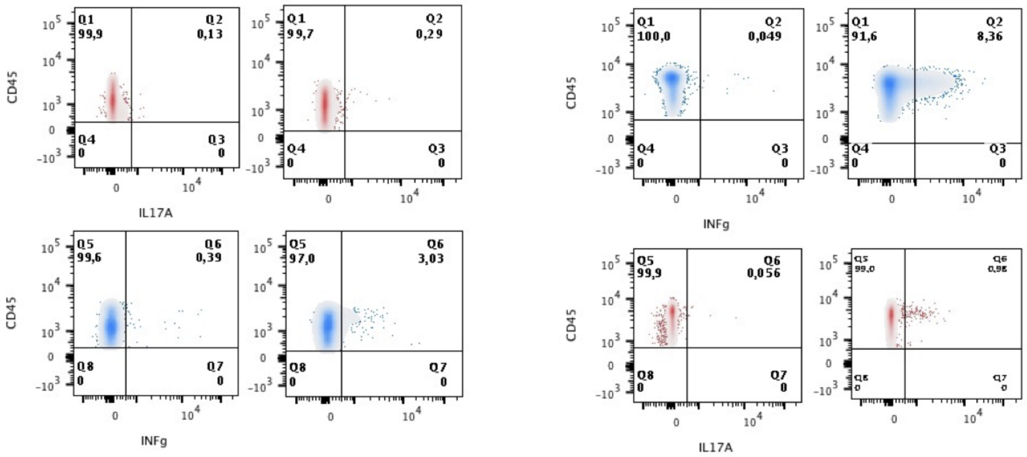

Figure S2

Gating strategy for CD45$^+$IL-17A$^+$ cells

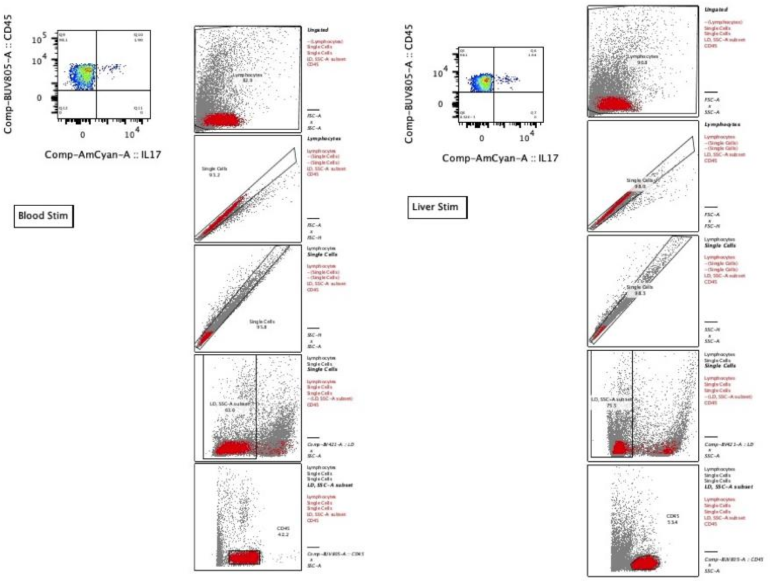

Supplement: Supplementary file 1 [file ijms-23-09773-s001.zip › ijms-1844249-supplementary.pdf]
